# Mundgesundheit bei Menschen mit Behinderung in Deutschland – eine Literaturübersicht

**DOI:** 10.1007/s00103-021-03352-7

**Published:** 2021-06-08

**Authors:** Andreas G. Schulte, Peter Schmidt

**Affiliations:** Abteilung für Behindertenorientierte Zahnmedizin, Universität Witten/Herdecke, Alfred-Herrhausen-Straße 50, 58448 Witten, Deutschland

**Keywords:** Kariesprävalenz, DMFT-Index, Zahnmedizin, Kinder und Jugendliche mit Behinderung, Erwachsene mit Behinderung, Caries prevalence, DMFT index, Dentistry, Children and adolescents with disability, Adults with disability

## Abstract

Zur Frage der Mundgesundheit bei Menschen mit Behinderung in Deutschland wurde die letzte Übersichtsarbeit im Jahr 2012 veröffentlicht. Dafür konnten lediglich 3 Feldstudien zur Kariesprävalenz bei Kindern, Jugendlichen oder Erwachsenen mit Behinderung identifiziert werden, die zwischen den Jahren 2000 und 2012 publiziert worden waren. Das Ziel der vorliegenden Übersichtsarbeit ist es, einen Überblick zum aktuellen Stand der Daten zur Mundgesundheit bei Menschen mit Behinderung in Deutschland zu geben und auf die Konsequenzen aufmerksam zu machen, die sich daraus ergeben. Es wurden sämtliche Publikationen und Abstracts eingeschlossen, die zwischen dem 01.01.2000 und dem 31.01.2021 publiziert bzw. zur Publikation angenommen worden waren.

In Deutschland wurden im eingeschlossenen Zeitraum 6 regionale Studien und 1 überregionale Studie bei Kindern und Jugendlichen mit Behinderung sowie 3 regionale Studien und 1 überregionale Studie bei Erwachsenen mit Behinderung in 4 Bundesländern durchgeführt. Darin wurde die Karieserfahrung mithilfe des dmft- bzw. DMFT-Index bestimmt. Es zeigte sich, dass Kinder mit Behinderung im Grundschulalter im Mittel eine deutlich höhere Karieserfahrung hatten als Kinder ohne Behinderung. Im Jugendalter trifft dies nur für Personen mit geistiger Behinderung zu. Bei Erwachsenen mit geistiger Behinderung wirkte sich die höhere Karieserfahrung so aus, dass insgesamt mehr Zähne extrahiert wurden als in der Allgemeinbevölkerung.

Daraus wird die Schlussfolgerung gezogen, dass die zahnärztliche und präventive Betreuung von Personen mit Behinderung weiter intensiviert werden muss. Dies kann u. a. durch die Berücksichtigung der zahnmedizinischen Versorgung von Menschen mit Behinderung in der Approbationsordnung Zahnmedizin und in den Curricula für das Fach Zahnmedizin an den einzelnen Universitätsstandorten erreicht werden.

## Abstract (en)

The last systematic review regarding oral health of persons with disability in Germany was published in 2012. For that review, only three field studies on caries prevalence of children, adolescents, or adults with disability published between 2000 and 2012 were identified. The aim of the present overview is to present the results of new studies and the evolving consequences. In this regard, all studies and abstracts that had been published or accepted for publication between 1 January 2000 and 31 January 2021 were included.

In this time interval, six regional studies and one supra-regional study were conducted in Germany in children and adolescents with disability. In addition, three regional studies and one supra-regional study were conducted in adults with disability. The studies had taken place in four of the 16 German federal states. Caries experience of the study participants was determined with the aid of a specific index. On average, children with disability and aged between 6 and 10 years had a higher caries experience than children without disability. In adolescents this difference is only true for those with intellectual disability. In adults with intellectual disability the higher caries experience yielded in a higher mean number of missing teeth compared to the general population.

In conclusion, preventive and dental care for persons with disability still has to be intensified. This goal may be achieved by adding dental care for persons with disability to the national dental curriculum and by integrating this discipline in the education of dental students in all universities where dentistry is taught.

## Einleitung

Die Tatsache, dass entsprechend den Angaben des Statistischen Bundesamts seit vielen Jahren die Zahl der Personen mit amtlich anerkannter Schwerbehinderung in Deutschland fast 8 Mio. beträgt [1], bedeutet nicht, dass alle Behinderungen mit einem Problem für die Mundgesundheit einhergehen müssen. Gemäß der UN-Behindertenrechtskonvention werden grundsätzlich 4 Arten von Behinderungen unterschieden: a) geistige Behinderung, b) körperliche Behinderung, c) psychische Behinderung und d) sensorische Behinderung [2]. Alle diese Behinderungen können nicht nur einzeln, sondern auch in unterschiedlichen Kombinationen vorliegen. In diesem Zusammenhang muss darauf hingewiesen werden, dass nicht alle Behinderungen von Geburt an bestehen oder in der frühen Kindheit auftreten, sondern zum größten Teil im Laufe des Lebens erworben werden.

In einem systematischen Review aus dem Jahr 2012 wurde gezeigt, dass in Deutschland die Mundgesundheit bei Menschen mit geistiger Behinderung schlechter ist als die der Allgemeinbevölkerung [3]. Hierfür konnten jedoch nur 3 Feldstudien ausgewertet werden. Bisher wurde noch von keiner zahnmedizinischen Fachgruppe ein Konsensus publiziert, welche Behinderung eine besondere Herausforderung für die Erhaltung der Mundgesundheit darstellt. Aus der klinischen Erfahrung heraus kann man versuchen, sich der Beantwortung dieser Frage zu nähern, indem in Bezug auf Personen mit Behinderung die folgenden 6 Kardinalfragen gestellt werden:

1. Ist die Person in der Lage, eigenverantwortlich in ausreichendem Maß die Mund- und Zahnpflege bei sich selbst durchzuführen?
2. Ernährt sich die Person sehr einseitig mit kohlenhydratreicher Nahrung?
3. Ist eine Kommunikation zwischen Person und Zahnarzt und somit der Aufbau eines Vertrauensverhältnisses zwischen beiden möglich?
4. Kann die Person bei zahnärztlichen Untersuchungen und Behandlungen kooperieren?
5. Hat der Zahnarzt genügend Fachkenntnisse, um die besonderen Aspekte der verschiedenen Behinderungen bei der zahnärztlichen Betreuung berücksichtigen zu können?
6. Ist die Person in der Lage ohne Unterstützung, aber ggfs. mit Hilfsmitteln, wie z. B. einem Rollstuhl, eine Zahnarztpraxis aufzusuchen?

Die Beantwortung dieser Fragen führt aus Sicht der Autoren der vorliegenden Publikation zu einer Liste an Beeinträchtigungen oder Behinderungen, die eine negative Auswirkung auf die Mundgesundheit haben können. Diese in Infobox 1 aufgeführte Liste erhebt nicht den Anspruch auf Vollständigkeit.

Da Karies weltweit die chronische Erkrankung mit der höchsten Prävalenz ist [4], beschäftigt sich die große Mehrheit der Studien zur Mundgesundheit mit der Prävalenz und dem Schweregrad von Karies. Aus nationalen Studien zur Mundgesundheit in Deutschland geht hervor, dass die Karieserfahrung bei 12-jährigen Kindern stark rückläufig ist und dass diese bei jüngeren Erwachsenen ebenfalls deutlich abgenommen hat, wenn auch nicht so ausgeprägt wie bei den Kindern [5, 6]. In diesen Studien wird jedoch nicht auf die Kariesprävalenz von Personen mit Behinderung eingegangen. Ziel dieser Übersichtsarbeit ist es, einen Überblick zum aktuellen Stand der Daten zur Mundgesundheit bei Menschen mit Behinderung in Deutschland zu geben und auf die Konsequenzen aufmerksam zu machen, die sich daraus ergeben.

## Material und Methode

Die Literaturrecherche für diese Übersichtsarbeit erfolgte in Anlehnung an die Methodik, die 2012 von Schulte angewandt und beschrieben wurde [3]. Es sollten Studien identifiziert werden, in denen die Prävalenz von Karies oder Parodontitis bei Menschen mit Behinderung ermittelt worden ist. Es wurden die diesbezüglichen Publikationen eingeschlossen, die zwischen dem 01.01.2000 und dem 31.01.2021 publiziert bzw. zur Publikation angenommen worden waren. Es wurde eine Onlinesuche in den Datenbanken Pubmed und Google Scholar durchgeführt. Zusätzlich wurde eine Handsuche in den deutschsprachigen Zeitschriften *Deutsche Zahnärztliche Zeitschrift, Oralprophylaxe, prophylaxe impuls, Quintessenz, Schweizer Monatsschrift für Zahnmedizin* und *Stomatologie* durchgeführt. Da die Zahl der entsprechenden Studien, die vollständig in Zeitschriften publiziert worden waren, sehr gering war, wurden auch Abstracts von Tagungsbeiträgen, die im genannten Zeitraum zu der hier behandelten Thematik veröffentlicht worden waren, berücksichtigt.

In Infobox 2 sind die deutschen und englischen Begriffe aufgeführt, mit denen diese Suche durchgeführt wurde. Die Entscheidung zum Ein- oder Ausschluss der Studien entsprechend der unten aufgeführten Kriterien erfolgte anhand der Überschriften und nach Sichtung der Abstracts der gefundenen Artikel.

### Einschlusskriterien für die Auswertung

Es wurden nur Studien einbezogen, in denen die Prävalenz von Karies oder Parodontitis von in Deutschland lebenden Personen mit Behinderung untersucht wurde. Dabei spielte es keine Rolle, welche Art von Behinderung die untersuchten Personen hatten oder zu welcher Altersgruppe sie gehörten. Es wurden nur Artikel berücksichtigt, die in englischsprachigen oder deutschsprachigen Zeitschriften mit Peer-Review-Verfahren publiziert wurden.

### Ausschlusskriterien für die Auswertung

Studien, in denen keine in Deutschland lebenden Personen mit Behinderung untersucht wurden, wurden für die Auswertung ausgeschlossen. Außerdem wurden Fallberichte und Fortbildungsartikel zur zahnärztlichen Versorgung von Menschen mit Behinderung nicht berücksichtigt. Studien bei Personen mit Demenz wurden ausgeschlossen. Obwohl Demenz nach Definition der UN-Behindertenrechtskonvention zu den in ihr definierten Formen von Behinderung zuzuordnen ist, wird in Deutschland die Gruppe der Personen mit Demenz in der Regel im Bereich der Seniorenzahnmedizin berücksichtigt. Außerdem wurden keine Studien berücksichtigt, die sich auf die Therapie von Patienten mit Behinderung, die z. B. in einer Universitätsklinik für Zahn‑, Mund- und Kieferheilkunde (ZMK-Heilkunde) im Wachzustand oder in Allgemeinanästhesie erfolgte, bezogen.

In der vorliegenden Publikation wird der Begriff „Förderschule“ für Schulen verwendet, in denen Schüler mit besonderem Förderbedarf wegen einer sensorischen, geistigen oder körperlichen Behinderung beschult werden.

## Ergebnisse

### Kinder und Jugendliche

Die Literatursuche ergab, dass in Deutschland im eingeschlossenen Zeitraum 6 regionale Studien und eine überregionale Studie zur Kariesprävalenz und Karieserfahrung von Kindern und Jugendlichen mit Behinderung durchgeführt wurden [7–13]. Diese Studien wurden in 3 Bundesländern, nämlich Nordrhein-Westfalen (NRW; [12, 13]), Thüringen [8–11] und Niedersachsen [11] durchgeführt.

In 2 Studien wurden Kariesprävalenz und Karieserfahrung bei 6‑ bis 10-jährigen Kindern, die Grund- und Förderschulen, bzw. bei 10- bis 14-Jährigen, die Haupt- und Förderschulen im Rhein-Erft-Kreis (NRW) besuchten, bestimmt [12, 13]. In einer der Thüringer Untersuchungen wurde die Karieserfahrung bei 6‑ bis 16-jährigen Schülern mit geistiger Behinderung, Körperbehinderung und sensorischer Behinderung ermittelt [10]. In einer weiteren Studie aus Thüringen wurde die Karieserfahrung von 6‑ bis 18-jährigen Schülern mit geistiger und körperlicher Behinderung oder schwerem Gehörschaden mit derjenigen von Schülern aus der Allgemeinbevölkerung verglichen [8]. Diese Parameter waren ebenfalls Gegenstand einer Studie, in der 6‑ bis 16-jährige Schüler mit geistiger Behinderung oder psychoemotionalen Störungen in je 3 Regionen bzw. Städten aus Thüringen und Niedersachsen untersucht wurden [11]. In der einzigen überregionalen Studie wurden die Kariesprävalenz und die Karieserfahrung bei Kindern und Jugendlichen im Alter von 12 bis 17 Jahren mit geistiger Behinderung, die im Jahr 2008 an den nationalen Sommerspielen von Special Olympics Deutschland teilnahmen, bestimmt [7]. Da nur in einem Teil der Studien neben der Gruppe der Kinder und Jugendlichen mit Behinderung auch Kinder und Jugendliche der Allgemeinbevölkerung berücksichtigt werden konnten, wurden auch die Untersuchungen, die im Auftrag der Deutschen Arbeitsgemeinschaft für Jugendzahnpflege (DAJ) erfolgten, mit herangezogen [14]. Die Studie von Schwerz et al. wird im Weiteren nicht berücksichtigt, weil dort die Werte für die Karieserfahrung zwar getrennt für Milchzähne und bleibende Zähne, aber nicht getrennt nach Altersgruppen ausgewiesen werden [9].

Aus den oben aufgeführten Studien wurden die Daten zur Kariesprävalenz im Milchgebiss von 6‑ und 7‑jährigen Kindern mit bzw. ohne Behinderung entnommen und in Tab. 1 aufgeführt. Die Beschränkung auf 6‑ und 7‑Jährige erfolgte, um einen Vergleich mit repräsentativen Daten der gleichen Altersgruppe der Allgemeinbevölkerung ziehen zu können. Aus den Angaben in der Tab. 1 ist klar ersichtlich, dass in dieser Altersgruppe bei Kindern mit Behinderung eine deutlich höhere Kariesprävalenz festgestellt wurde als bei Kindern der Allgemeinbevölkerung.

**Table 1:** 

Studie	Region	Untersuchungsjahr	Art der Behinderung	Karieserfahrung (dmft > 0) (%)	95 % KI (%)
*Kinder mit Behinderung*					
Dziwak et al. 2017 [10]	Thüringen	2010/2011	GB, KB; SB	65,7	53,7–75,9
Schüler et al. 2019 [11]	Thüringen	2010/2011	GB, PES	72,7	k. A.
Schüler et al. 2019 [11]	Niedersachsen	2010/2011	GB, PES	52,6	k. A.
Schmidt et al. 2020 [12]	NRW	2010/2011	GB, SB, KB, PES	54,3	39,7–68,8
Schmidt et al. 2020 [12]	NRW	2015/2016	GB, SB, KB, PES	55,1	53,9–56,3
*Kinder ohne Behinderung*					
Schmidt et al. 2020 [12]	NRW	2010/2011	OB	41,8	41,3–42,3
Schmidt et al. 2020 [12]	NRW	2015/2016	OB	41,9	41,3–42,4
TEAM DAJ 2017 [14]	Deutschland	2015/2016	OB	43,6	k. A.

*DAJ* Deutsche Arbeitsgemeinschaft für Jugendzahnpflege, *dmft* Kariesindex für das Milchgebiss („decayed, missing, filled, tooth“), *GB* geistige Behinderung, *k.* *A.* keine Angabe, *KB* körperliche Behinderung, *KI* Konfidenzintervall, *NRW* Nordrhein-Westfalen; *OB* ohne Behinderung, *PES* psychoemotionale Störung, *SB* sensorische Behinderung

In Bezug auf die Karieserfahrung im Milchgebiss lässt sich feststellen, dass nur in 2 Studien sowohl Kinder mit Behinderung als auch solche ohne Behinderung in derselben Region untersucht wurden [8, 12]. Auch hier zeigt sich, dass bei Kindern mit Behinderung eine deutlich höhere Karieserfahrung vorliegt als bei Kindern ohne Behinderung (Tab. 2). Vergleiche mit nationalen Daten der Allgemeinbevölkerung sind hier nicht möglich, weil es diesbezüglich keine Studien in der Altersgruppe der 6‑ bis 11-jährigen Kinder gibt. Eine einzige Studie ging der Frage nach, ob die Karieserfahrung bei Kindern mit Behinderung im Verlauf von mehreren Jahren in derselben Region und derselben Altersgruppe rückläufig war [12]. Dort blieb der mittlere dmft-Wert (dmft = Kariesindex für das Milchgebiss) bei 6‑ bis 10-jährigen Kindern mit Behinderung in den Schuljahren 2010/2011 und 2015/2016 mit jeweils 2,1 unverändert.

**Table 2:** 

Studie	Untersuchungsjahr(e)	Alter in Jahren	Art der Behinderung	Kariöse Zähne (mittlerer dt)	Fehlende Zähne (mittlerer mt)	Gefüllte Zähne (mittlerer ft)	Karieserfahrung (mittlerer dmft)
*Kinder mit Behinderung*							
Hempel et al. 2015 [8]	2010/2011	6–12	GB, SB und KB	0,9	0,4	0,9	2,3
Dziwak et al. 2017 [10]	2010/2011	6–11	GB	0,9	0,4	0,8	2,1
Dziwak et al. 2017	2010/2011	6–11	KB	1,3	0,2	0,8	2,3
Dziwak et al. 2017 [10]	2010/2011	6–11	SB	1,0	0,2	0,9	2,1
Schüler et al. 2019 [11]	2010/2011 (Niedersachsen)	6–11	GB	1,8	0,3	0,3	2,4
Schüler et al. 2019 [11]	2010/2011 (Thüringen)	6–11	GB	0,9	0,4	0,8	2,1
Schüler et al. 2019 [11]	2010/2011	6–11	PES	1,4	0,0	1,1	2,5
Schmidt et al. 2020[12]	2010/2011	6–10	GB, SB, KB, PES	0,6	0,4	1,1	2,1
Schmidt et al. 2020 [12]	2015/2016	6–10	GB, SB, KB, PES	0,9	0,5	0,7	2,1
*Kinder ohne Behinderung*							
Hempel et al. 2015 [10]	2010/2011	6–12	OB	0,5	0,1	1,0	1,6
Schmidt et al. 2020 [12]	2010/2011	6–10	OB	0,7	0,2	0,6	1,5
Schmidt et al. 2020 [12]	2015/2016	6–10	OB	0,7	0,2	0,9	1,8

*DMTF* Kariesindex für das Milchgebiss („decayed, missing, filled, tooth“), *dt* „decayed teeth“, *ft* „filled teeth“, *GB* geistige Behinderung, *KB* körperliche Behinderung, *mt* „missing teeth“, *OB* ohne Behinderung, *PES* psychoemotionale Störung, *SB* sensorische Behinderung

Ein etwas differenzierteres Bild zeigt sich bei Betrachtung der Werte für die bleibenden Zähne der Kinder und Jugendlichen im Alter zwischen 10 und 18 Jahren (Tab. 3). In den hier aufgeführten Studien bewegten sich die mittleren DMFT-Werte (Kariesindex für das bleibende Gebiss) für 4 verschiedene Altersgruppen in einer Spannbreite von 0,8 bis 3,0. Aus diesen Studien lassen sich 2 Aspekte eindeutig ablesen: 1) In Nordrhein-Westfalen (NRW) waren die mittleren DMFT-Werte bei Kindern und Jugendlichen mit Behinderung in den Schuljahren 2010/2011 und 2015/2016 unverändert [13] und 2) Kinder und Jugendliche mit geistiger Behinderung haben eine deutlich höhere Karieserfahrung als solche mit Körperbehinderung [10].

**Table 3:** 

Studie	Untersuchungsjahr	Alter in Jahren	Art der Behinderung	Kariöse Zähne (mittlerer DT)	Fehlende Zähne (mittlerer MT)	Gefüllte Zähne (mittlerer FT)	Karieserfahrung (mittlerer DMFT)
*Kinder und Jugendliche mit geistiger Behinderung*							
Bissar et al. 2010 [7]	2008	12–17	GB	0,7	0,2	1,4	2,3
Hempel et al. 2015 [8]	2010/2011	13–18	GB, KB, SB	0,5	0,3	1,3	1,9
Dziwak et al. 2017 [10]	2010/2011	12–16	SB	0,6	0,2	1,3	2,1
Dziwak et al. 2017 [10]	2010/2011	12–16	KB	0,3	0,1	0,8	1,1
Dziwak et al. 2017 [10]	2010/2011	12–16	GB	0,8	0,2	1,4	2,4
Schüler et al. 2019 [11]	2010/2011	12–16	PES	1,6	0,0	1,4	3,0
Schüler et al. 2019 [11]	2010/2011 (Thüringen)	12–16	GB	0,8	0,2	1,4	2,4
Schüler et al. 2019 [11]	2010/2011 (Niedersachsen)	12–16	GB	1,7	0,2	0,9	2,8
Schmidt et al. 2021 [13]	2010/2011 (NRW)	10–14	GB, SB, KB, PES	0,3	0,1	0,4	0,8
Schmidt et al. 2021 [13]	2015/2016 (NRW)	10–14	GB, SB, KB, PES	0,3	0,1	0,4	0,8
*Kinder und Jugendliche ohne geistige Behinderung*							
Hempel et al. 2015 [8]	2010/2011	13–18	OB	0,5	0,1	1,6	2,1

*DMFT* Kariesindex für das bleibende Gebiss („decayed, missing, filled, tooth“), *DT* „decayed teeth“, *FT* „filled teeth“, *GB* geistige Behinderung, *KB* körperliche Behinderung, *MT* „missing teeth“, *NRW* Nordrhein-Westfalen, *OB* ohne Behinderung, *PES* psychoemotionale Störung, *SB* sensorische Behinderung

Da Fissurenversiegelungen (FV) einen wichtigen Baustein in der Kariesprävention von Kindern und Jugendlichen darstellen, sollen an dieser Stelle die diesbezüglich wichtigsten Ergebnisse aus den oben aufgeführten Studien präsentiert werden. Bissar et al. (2010) stellten fest, dass bei 47,5 % der von ihnen untersuchten Kinder und Jugendlichen mit geistiger Behinderung mindestens ein Zahn fissurenversiegelt war [7]. Der mittlere DMFT-Wert betrug bei den Personen mit mindestens einer FV 1,72 und bei denjenigen ohne FV 2,85 [7]. Schmidt et al. (2021) berichteten, dass sich der Anteil der Kinder und Jugendlichen mit Behinderung im Schuljahr 2010/2011 auf 36,3 % und im Schuljahr 2015/2016 auf 52,1 % belief [13]. Die mittleren DMFT-Werte betrugen 0,56 bzw. 0,51 (Gruppe der Personen mit mindestens einer FV) und 0,90 bzw. 1,02 (Gruppe der Personen ohne FV; [13]). Hempel et al. (2015) beobachteten, dass in der Gruppe der Kinder und Jugendlichen mit Behinderung der Anteil der Personen mit mindestens einer FV 45,5 % und in der Gruppe der Personen ohne Behinderung jedoch 75,6 % betrug [8]. Einen ähnlichen Unterschied konnten Dziwak et al. (2017) beim Vergleich von Kindern und Jugendlichen mit geistiger Behinderung auf der einen Seite (44,3 %) und Kindern und Jugendlichen mit Körperbehinderung auf der anderen Seite feststellen (75,6 %; [10]).

### Erwachsene

Studien zur Kariesprävalenz und Karieserfahrung bei Menschen mit Behinderung wurden in Deutschland in der Gruppe der Erwachsenen nur bei Menschen mit geistiger Behinderung durchgeführt. Untersucht wurden 2 unterschiedliche Arten von Kollektiven. Dabei handelt es sich zum einen um Personen, die in speziellen Werkstätten für Menschen mit geistiger Behinderung beschäftigt sind, und zum anderen um Athleten mit geistiger Behinderung, die an den nationalen Sommerspielen von Special Olympics Deutschland teilnahmen.

Seit dem Jahr 2000 wurden die Ergebnisse von 3 Studien publiziert, die bei Erwachsenen mit geistiger Behinderung in den Werkstätten, in denen diese Personen beschäftigt waren, durchgeführt worden waren [15–17]. Diese Studien wurden zwischen 2007 und 2018 in Baden-Württemberg, Sachsen und Nordrhein-Westfalen [15–17] durchgeführt. Eine weitere Studie wurde 2008 bei Athleten mit geistiger Behinderung im Rahmen einer nationalen Sportveranstaltung von Special Olympics Deutschland durchgeführt [18]. In Tab. 4 sind die mittleren Werte für die Zahl der kariösen (DT), fehlenden (MT) und gefüllten Zähne (FT) aus diesen Studien aufgeführt. Diese Tabelle enthält auch die entsprechenden Werte für die 35- bis 44-Jährigen aus der deutschen Allgemeinbevölkerung, die im Rahmen der Deutschen Mundgesundheitsstudien DMS IV und DMS V in den Jahren 2005 und 2014 durchgeführt wurden [6, 19]. Auch wenn die Altersgruppen dieser Studien nicht ganz identisch sind, zeigt sich sehr deutlich, dass bei Menschen mit geistiger Behinderung der mittlere MT-Wert, d. h. die mittlere Anzahl extrahierter Zähne, wesentlich höher ist als bei den Menschen der Allgemeinbevölkerung.

**Table 4:** 

Studie	Untersuchungsjahr	Alter in Jahren	Art der Behinderung	Kariöse Zähne (mittlerer DT)	Fehlende Zähne (mittlerer MT)	Gefüllte Zähne (mittlerer FT)	Karieserfahrung (mittlerer DMFT, (95 %-KI))
*Erwachsene mit geistiger Behinderung*							
Schulte et al. 2010 [18]	2008	Im Mittel 30,8	GB	0,9	2,9	5,6	9,4 (k. A.)
Schulte et al. 2013 [15]	2007/2008	Im Mittel 35,6	GB	1,9	5,7	4,6	12,3 (11,6–12,9)
Wellkamp et al. 2018 [16]	2015/2016	Im Mittel 41,0	GB	1,1	6,6	4,9	12,7 (k. A.)
Schulte et al. 2020 [17]	2017/2018	Im Mittel 35,5	GB	0,5	4,4	4,8	9,7 (8,2–11,1)
*Erwachsene der Allgemeinbevölkerung*							
Micheelis und Schiffner 2006^a^ [19]	2005	35–44	OB	0,5	2,4	11,7	14,5 (k. A.)
Jordan und Micheelis 2016^b^ [6]	2014	35–44	OB	0,5	2,1	8,6	11,2 (k. A.)

*DMFT* Kariesindex für das bleibende Gebiss („decayed, missing, filled, tooth“), *DT* „decayed teeth“, *FT* „filled teeth“, *GB* geistige Behinderung, *k.* *A.* keine Angabe, *KI* Konfidenzintervall, *MT* „missing teeth“, *OB* ohne Behinderung

^a^ repräsentative Deutsche Mundgesundheitsstudie DMS IV

^b^ repräsentative Deutsche Mundgesundheitsstudie DMS V

## Diskussion

### Studienpopulation

Die vorliegende Literaturübersicht zeigt, dass in Deutschland Studien zur Prävalenz von Karies bei Menschen mit Behinderung nur in sehr geringer Zahl und fast nur in umschriebenen Regionen von 4 Bundesländern durchgeführt wurden. Lediglich in den Studien, die bei Athleten mit geistiger Behinderung im Rahmen von nationalen Sommerspielen von Special Olympics Deutschland durchgeführt wurden, stammten die untersuchten Personen aus allen Teilen Deutschlands [7, 18]. Es fällt außerdem auf, dass nur in 2 Studien die Kariesprävalenz sowohl von Kindern und Jugendlichen mit Behinderung als auch von solchen der Allgemeinbevölkerung in derselben Region bestimmt wurde [8, 12]. Die Ergebnisse der anderen in dieser Arbeit aufgeführten Studien können nur mit den Ergebnissen der nationalen Studien verglichen werden, die im Auftrag der DAJ bei Kindern und Jugendlichen durchgeführt wurden [5, 14] oder die im Auftrag von Bundeszahnärztekammer und Kassenzahnärztlicher Bundesvereinigung bei 12-jährigen Kindern und Erwachsenen durchgeführt wurden [6]. Es erscheint dringend notwendig, bei kariesepidemiologischen Untersuchungen bei Kindern und Jugendlichen mit Behinderung in Zukunft immer eine vergleichbare Kontrollgruppe aus der Allgemeinbevölkerung mit zu untersuchen. Ansonsten besteht die Gefahr, dass Studien ohne Kontrollgruppen in internationalen systematischen Reviews zu der Frage nach der Karieserfahrung von Menschen mit Behinderung nicht eingeschlossen werden. Dies zeigte sich z. B. 2019, als ein internationales systematisches Review publiziert wurde, das 23 Studien zur Karieserfahrung von Kindern mit geistiger Behinderung aus vielen Ländern der Welt enthielt, aber leider keine Studie aus Deutschland [20].

Wegen der allgemeinen Schulpflicht lässt sich die Altersgruppe der Kinder und Jugendlichen gut in den Schulen erreichen. Allerdings ist die Zahl der Kinder und Jugendlichen mit Behinderung im Vergleich zur Allgemeinbevölkerung sehr klein. Daraus resultiert, dass bei der Untersuchung von Kindern und Jugendlichen in Schulen einer Region die für die Auswertung zur Verfügung stehende Fallzahl sehr gering ist. Dies hat wiederum zur Folge, dass in den Untersuchungen über die Karieserfahrung von Kindern und Jugendlichen mehrere Altersstufen zusammengefasst werden müssen (Tab. 2 und 3). Im Gegensatz dazu empfiehlt die Weltgesundheitsorganisation (WHO) kariesepidemiologische Untersuchungen vor allem in 3 Altersgruppen durchzuführen: 5‑ und 6‑Jährige, 12-Jährige und 35- bis 44-Jährige [21]. Zu diesen Altersgruppen liegen für die Allgemeinbevölkerung aus Deutschland mehrere nationale Studien vor, die die Entwicklung der Kariesprävalenz seit den 1990er-Jahren dokumentieren [5, 6, 14, 19].

In der Altersgruppe der Erwachsenen mit Behinderung lassen sich oralepidemiologische Untersuchungen am einfachsten in Werkstätten für Beschäftigte mit geistiger Behinderung und bei den Sportveranstaltungen von Special Olympics durchführen. Special Olympics versteht sich ausdrücklich als Organisation für Menschen mit geistiger Behinderung. Deshalb sind bei den Sportveranstaltungen von Special Olympics Menschen mit anderen Behinderungen, wie z. B. Blindheit, Gehörlosigkeit oder Querschnittslähmung, nur dann vertreten, wenn gleichzeitig eine geistige Behinderung vorliegt. Repräsentative oralepidemiologische Untersuchungen bei Menschen mit Blindheit, Gehörlosigkeit oder Querschnittslähmung lassen sich nach Ansicht der Autoren der vorliegenden Übersichtsarbeit nur durchführen, indem man die Mitglieder von entsprechenden Interessengruppen (z. B. Deutscher Blinden- und Sehbehindertenverband e. V. oder Deutscher Verband der Gehörlosen e. V.) bittet, sich daran zu beteiligen.

Dennoch wäre es wünschenswert, in mehrjährigen Abständen, z. B. alle 5 bis 7 Jahre, oralepidemiologische Untersuchungen bei Menschen mit den verschiedenen Arten von Behinderung in allen Altersstufen durchzuführen. Gleichzeitig sollte auch eine altersmäßig entsprechende Kontrollgruppe aus der Allgemeinbevölkerung untersucht werden. Außerdem wäre es hilfreich, diese Studien in allen deutschen Bundesländern durchzuführen, um ggfs. vorhandene regionale Unterschiede in Bezug auf die Mundgesundheit und die zahnmedizinische Versorgung zu erkennen.

Schließlich sollte auch bedacht werden, dass es allein in der Gruppe der Personen mit geistiger Behinderung sehr viele unterschiedliche Subgruppen gibt (z. B. Down-Syndrom, die Autismus-Spektrum-Störungen, Cerebralparese, Fragiles-X-Syndrom, Rett-Syndrom, Angelman-Syndrom oder Williams-Beuren-Syndrom), bei denen aus sehr unterschiedlichen Gründen die Mundgesundheit beeinträchtigt sein kann. Angehörige dieser Subgruppen können im Rahmen der kollektiven Untersuchung in Schulen, Werkstätten oder bei Sportveranstaltungen in der Regel nicht identifiziert werden. Es wäre jedoch sehr sinnvoll, nicht nur über die Mundgesundheit der Gesamtgruppe der Menschen mit geistiger Behinderung Informationen zu haben, sondern auch über spezielle Subgruppen.

Verschiedene Gründe bestehen, warum solche Studien bisher nicht durchgeführt wurden. Zum einen fehlt es in den deutschen Universitätskliniken für ZMK-Heilkunde an personellen Ressourcen, um derartige Studien zu planen und zu begleiten. Dies lässt sich z. B. auch daran erkennen, dass es an den 31 deutschen Universitätskliniken für ZMK-Heilkunde nur einen Lehrstuhl für Behindertenorientierte Zahnmedizin sowie nur noch 3 offizielle Professuren für Kinderzahnheilkunde gibt. Zum anderen ist es sehr schwer, für diese Studien die finanzielle Ausstattung zur Durchführung und Auswertung zu erhalten. In diesem Zusammenhang können die Autoren berichten, dass vonseiten sehr vieler Organisationen, die jeweils die Interessen von Menschen mit einer bestimmten Behinderungsart vertreten, ein hohes Interesse an Studien zum Thema Mundgesundheit besteht. Jedoch fehlt es auch diesen Organisationen an der finanziellen Ausstattung zur Unterstützung derartiger Studien.

### Kariesprävalenz und Karieserfahrung

Aus den Angaben zu den Kariesprävalenzraten der 6‑ und 7‑Jährigen (Tab. 1) lässt sich ablesen, dass bei Kindern mit Behinderung im Vergleich zur Allgemeinbevölkerung eine deutlich höhere Kariesbelastung vorliegt. Diese Kinder besuchen in der Regel die erste Klasse einer Schule und haben die kariösen Zähne zum größten Teil bereits in den Jahren vor der Einschulung erworben. Dies bedeutet, dass Kinder mit Behinderung schon sehr früh, d. h. eigentlich vom ersten vorhandenen Milchzahn an, eine kariespräventive Betreuung benötigen. In einer Befragung von Personen, die einen Familienangehörigen mit Downsyndrom haben, zeigte sich, dass nur 10 % der Personen mit Downsyndrom vor ihrem ersten Geburtstag einem Zahnarzt vorgestellt wurden [22]. Die Voraussetzungen hierfür sind in den letzten Jahren etwas verbessert worden, weil Zahnärzte nun bei allen Kindern im Alter von 0 bis 71 Monaten mehrmals präventive Leistungen zulasten der gesetzlichen Krankenkassen erbringen können. Hinzu kommt, dass seit dem 01.08.2018 gesetzlich krankenversicherte Personen aus allen Altersstufen, denen ein Pflegegrad zuerkannt wurde oder die Eingliederungshilfe beziehen, Anspruch auf Beratungen und Unterweisungen zur Mundpflege haben. Deshalb ist die Information, dass z. B. sehr vielen Kindern mit Down-Syndrom bereits im Kindesalter ein Pflegegrad zuerkannt wird [22], sehr wichtig.

Aus den in der Tab. 2 angegeben dmft-Werten lässt sich ablesen, dass Schulkinder mit Behinderung eine deutlich höhere Karieserfahrung im Milchgebiss haben als solche ohne Behinderung [10, 12]. Zahnmedizinisch präventive Leistungen müssen unbedingt auch in dieser Altersphase bei Kindern mit Behinderung erbracht werden, weil dann der Durchbruch der ersten bleibenden Zähne beginnt. Eine dieser Präventionsleistungen besteht aus Fissurenversiegelungen, auf die weiter unten noch näher eingegangen wird. Außerdem können die Kinder davon profitieren, dass in den Zahnarztpraxen wegen des erhöhten Kariesrisikos die Fluoridierung der Zähne nicht nur zweimal, sondern viermal pro Jahr durchgeführt werden kann. Parallel dazu sollten die Fluoridierungen auch im Rahmen der Gruppenprophylaxe durch den Zahnärztlichen Dienst der Gesundheitsämter bzw. durch Arbeitsgemeinschaften für Zahnpflege erbracht werden [12]. Ein weiterer Baustein der präventiven Maßnahmen besteht in den Beratungen und Anleitungen zur Mundpflege, die bei gesetzlich versicherten Kindern und Jugendlichen im Rahmen der Individualprophylaxe und zusätzlich ein weiteres Mal pro Kalenderhalbjahr erbracht werden können, wenn ein Pflegegrad vorliegt oder Eingliederungshilfe bezogen wird.

In Bezug auf das bleibende Gebiss von Kindern und Jugendlichen mit Behinderung kann die Aussage, dass diese eine höhere Karieserfahrung als Kinder und Jugendliche ohne Behinderung haben, anhand der vorliegenden Daten nicht so pauschal getroffen werden (Tab. 3). Hempel et al. (2015) fanden in diesen beiden Gruppen sehr ähnliche mittlere DMFT-Werte [8]. Schaut man sich die Daten von Dziwak et al. (2017) genauer dann, wird ein großer Unterschied in Bezug auf die Karieserfahrung der Probanden mit unterschiedlichen Behinderungen deutlich [10]. Dort wurde bei Kindern und Jugendlichen mit geistiger Behinderung ein mehr als doppelt so hoher mittlerer DMFT-Wert (2,4) wie bei den Kindern und Jugendlichen mit Körperbehinderung (1,1) festgestellt [10]. Der Mittelwert von 2,4 ist sehr ähnlich dem Mittelwert von 2,3, welchen Bissar et al. (2010; [7]) bei fast gleichaltrigen Jugendlichen mit geistiger Behinderung ermittelten. Dies zeigt sehr deutlich, dass der mittlere DMFT-Wert bei einem Kollektiv von Kindern und Jugendlichen mit Behinderung ab einem Alter von > 10 Jahren sehr davon beeinflusst wird, ob es nur Personen mit geistiger Behinderung oder auch solche mit Körperbehinderung umfasst. Dazu passt, dass in dem von Hempel et al. (2015) untersuchten Kollektiv neben Personen mit geistiger auch jene mit körperlicher oder sensorischer Behinderung enthalten sind [8]. An dieser Stelle soll auch darauf hingewiesen werden, dass Kinder und Jugendliche mit psychoemotionalen Störungen ein ähnlich hohes Kariesrisiko aufweisen wie die entsprechende Altersgruppe mit geistiger Behinderung [11].

Im Ergebnisteil wurde dargestellt, dass Fissurenversiegelungen (FV) auch bei Kindern und Jugendlichen einen wesentlichen Beitrag zur Kariesprävention leisten. Allerdings zeigen die Prävalenzzahlen zu FV, dass der Anteil der Kinder und Jugendlichen mit Behinderung, die über mindestens einen bleibenden Zahn mit FV verfügen, deutlich geringer ist als bei gleichaltrigen Personen der Allgemeinbevölkerung [8]. Deshalb müssen Zahnärzte und Eltern informiert und motiviert werden, Kinder mit Behinderung in demselben Ausmaß mit Fissurenversiegelungen zu versorgen wie Kinder ohne Behinderung.

Die Karieserfahrung wurde nur in sehr wenigen Studien bei Erwachsenen mit geistiger Behinderung untersucht (Tab. 4). In keiner dieser Studien konnte eine Kontrollgruppe aus gleichaltrigen Erwachsenen ohne Behinderung einbezogen werden. Deshalb sind diesbezüglich nur Vergleiche mit den nationalen Deutschen Mundgesundheitsstudien IV und V möglich. Eine Einschränkung in diesem Zusammenhang besteht darin, dass die Zahl der Personen im Alter von 35 bis 44 Jahren, die in den Werkstätten untersucht wurden, sehr gering war und nicht gut zum Vergleich mit den entsprechenden Daten aus den DMS-Studien geeignet ist. Deshalb wurden in Tab. 4 der mittlere DMFT-Wert für jeweils alle Personen, die in den 3 Werkstätten untersucht wurden, sowie das entsprechende mittlere Alter angegeben.

Obwohl die in der Tab. 4 aufgeführten Kohorten nur annähernd vom Alter her zusammenpassen, lässt sich doch eindeutig feststellen, dass bei Erwachsenen mit geistiger Behinderung viel mehr fehlende Zähne zu verzeichnen sind als bei Erwachsenen der Allgemeinbevölkerung. Aus dem Vergleich der mittleren DMFT-Werte und den dazugehörigen 95 %-Konfidenzintervallen der beiden Studien von Schulte et al. (2013) und Schulte et al. (2020) lässt sich ablesen, dass die mittlere Karieserfahrung bei Erwachsenen mit geistiger Behinderung leicht rückläufig ist [15, 17]. Diese Aussage geschieht jedoch unter dem Vorbehalt, dass es sich hierbei nur um regionale Studien handelt, die zwar in einem Abstand von 10 Jahren, aber in 2 unterschiedlichen Bundesländern durchgeführt wurden. Trotz der rückläufigen Zahlen muss die präventive Versorgung von Menschen mit geistiger Behinderung intensiviert werden. Ein guter Schritt auf diesem Weg ist die seit 2018 bestehende Möglichkeit für Zahnärzte, auch bei gesetzlich krankenversicherten Erwachsenen mit anerkanntem Pflegegrad oder mit Bezug von Eingliederungshilfe präventive Leistungen zu erbringen. Die besonderen Vorgehensweisen, die bei der Erbringung zahnmedizinisch präventiver Leistungen vor allem bei Patienten mit geistiger Behinderung erforderlich sind, wurden in 2 Fachbeiträgen beschrieben [23, 24].

### Herausforderungen und Ausblick

Leider wurden im Rahmen der Literatursuche keine Studien zur Prävalenz von Parodontitis bei Menschen mit Behinderung gefunden. Eine der Herausforderungen bei diesen Studien ist, dass hierfür das Messen der Sondierungstiefen an mehreren Stellen pro Zahn erforderlich ist. Dieser Vorgang wird von sehr vielen Personen mit geistiger Behinderung im Wachzustand nicht in ausreichendem Maß oder gar nicht toleriert. Dennoch sollten Experten versuchen, Lösungen zu finden, wie man das Vorliegen von Parodontitis bei dieser Bevölkerungsgruppe bestimmen kann.

Ein weiteres Feld der zahnmedizinischen Versorgung von Menschen mit Behinderung betrifft die Notwendigkeit, dass vor allem ein Teil der Menschen mit geistiger Behinderung darauf angewiesen ist, zumindest einmal im Leben eine zahnärztliche Therapie unter Allgemeinanästhesie zu erhalten. Bei Menschen mit Down-Syndrom beträgt dieser Anteil ca. 40 % [22]. Die Autoren müssen aufgrund ihrer klinischen Tätigkeit in der Versorgung von Patienten mit Behinderung feststellen, dass es diesbezüglich noch große Defizite gibt, die gelöst werden müssen. Dazu zählt u. a. die Tatsache, dass die Zahl und Kapazität der Zahnärzte bzw. klinischen Einrichtungen, die zahnmedizinische Behandlungen in Allgemeinanästhesie anbieten, derzeit nicht ausreicht. Dies gilt in ganz besonderem Maß für Patienten, bei denen eine Therapie in Allgemeinanästhesie nicht ambulant durchgeführt werden kann, sondern wegen eines hohen allgemeinmedizinischen Risikos stationär in einem Krankenhaus erfolgen muss. Aus Platzgründen kann diese Problematik hier nicht ausführlicher beleuchtet werden.

Eine gute zahnmedizinische und präventiv orientierte Versorgung von Menschen mit Behinderung hängt jedoch nicht nur davon ab, welche Leistungen der Katalog der gesetzlichen Krankenversicherungen enthält. Vielmehr muss in diesem Zusammenhang darauf aufmerksam gemacht werden, dass eine gute zahnmedizinische Versorgung von Patienten mit Behinderung auch davon abhängt, dass diese auch Teil des Studiums für angehende Zahnärzte ist. Vor einigen Jahren berichteten Zahnärzte aus Thüringen und Baden-Württemberg im Rahmen von 2 Fragebogenstudien, dass sie im Studium nicht oder nur unzureichend auf die zahnmedizinische Versorgung von Patienten mit Behinderung vorbereitet wurden [25, 26]. Leider werden weder in der alten noch in der neuen Approbationsordnung Zahnmedizin Unterrichtsveranstaltungen in Zahnmedizin für Patienten mit Behinderung gefordert [27].

Zusammenfassend zeigen die hier präsentierten Studien und Ausführungen, dass eine jahrzehntelange kompetente zahnmedizinische Betreuung bei Menschen mit Behinderung erforderlich ist, um bei ihnen die Zahn- und Mundgesundheit im Verlauf des Lebens zu erhalten. Diese Bemühungen werden immer noch durch zahlreiche Barrieren erschwert, auch wenn in den letzten Jahren wichtige Schritte in die richtige Richtung unternommen wurden.

#### Infobox 1 Arten von Behinderung, die einen negativen Einfluss auf die Mundgesundheit haben können

- Geistige Behinderung
- Psychische Behinderung
- Demenz
- Kognitive Beeinträchtigungen, z. B. infolge eines Apoplexes, einer Meningitis oder eines Schädelhirntraumas
- Mehrfachbehinderung (z. B. das gemeinsame Vorliegen einer Körperbehinderung und einer kognitiven Behinderung)
- Blindheit oder schwere Sehbehinderung
- Gehörlosigkeit oder schwere Hörbehinderung
- Angewiesensein auf einen Rollstuhl
- Fehlende oder teilweise bzw. vollständig gelähmte Hände oder Arme

#### Infobox 2 Begriffe, mit denen in unterschiedlichen Kombinationen Studien zur Mundgesundheit bei Menschen mit Behinderung in Deutschland in Pubmed, in Google Scholar (deutsch) oder per Hand gesucht wurden

*Deutsche Suchbegriffe*:

Mundgesundheit

Zahngesundheit

Kariesprävalenz

Karieserfahrung

Parodontitisprävalenz

DMFT Index

Behinderung

Geistige Behinderung

Mehrfach-Behinderung

Körperbehinderung

Kinder

Jugendliche

Erwachsene

Deutschland

*Englische Suchbegriffe*:

Oral health

Dental health

Caries prevalence

Caries experience

Prevalence periodontitis

DMFT Index

Dental health survey

Disability

Intellectual disability

Learning disability

Children

Adolescents

Adults

Germany
